# Mindfulness as a Path to Freedom from Internet Addiction in Adolescents: A Narrative Review

**DOI:** 10.7759/cureus.72544

**Published:** 2024-10-28

**Authors:** Priyadarsini Samanta, Ipsa Mohapatra, Rituparna Mitra, Jayanti Mishra, Pranab Mahapatra, Nirmal K Mohakud, Jigyansa I Pattnaik, Manas Ranjan Behera, Pranati Nanda

**Affiliations:** 1 Department of Physiology, Kalinga Institute of Medical Sciences, Bhubaneswar, IND; 2 Department of Community Medicine, Kalinga Institute of Medical Sciences, Bhubaneswar, IND; 3 Department of Research and Development, Kalinga Institute of Medical Sciences, Bhubaneswar, IND; 4 Department of Physiology, All India Institute of Medical Sciences, Bhubaneswar, IND; 5 Department of Psychiatry, Kalinga Institute of Medical Sciences, Bhubaneswar, IND; 6 Department of Pediatric Medicine, Kalinga Institute of Medical Sciences, Bhubaneswar, IND; 7 Department of Child and Adolescent Psychiatry, Kalinga Institute of Medical Sciences, Bhubaneswar, IND; 8 School of Public Health, Kalinga Institute of Industrial Technology (KIIT) Deemed to be University, Bhubaneswar, IND

**Keywords:** adolescents, emotional regulation, executive functioning, impulsive behavior, internet addiction disorder, mindfulness, psychological well-being

## Abstract

In recent years, Internet Addiction Disorder (IAD) has become a major mental health concern among adolescents, with detrimental effects on social relationships, academic performance, and emotional well-being. The focus of mindfulness-based interventions (MBIs) on improving self-awareness, emotional regulation, and cognitive flexibility has drawn interest in MBIs as possible therapeutic approaches for treating IAD. The purpose of this narrative review is to investigate how mindfulness interventions affect teenagers who are experiencing IAD. The review summarizes research findings from multiple studies that look at how well MBIs work to promote healthier online behavior, improve mental health outcomes, and cut down on excessive internet use. According to recent research, adolescents who engage in mindfulness practices are better able to control their compulsive internet usage habits, resist impulsive impulses, and spend less time on the internet. Furthermore, mindfulness practices like breathing exercises, body scanning, and meditation may help reduce stress, anxiety, and depression, which are the common comorbidities linked to IAD. The review ends by emphasizing the necessity of more research on the long-term impacts of MBIs on IAD, the incorporation of mindfulness into school-based initiatives, and the significance of developing interventions that are specific to the developmental needs of teenagers. All things considered, mindfulness intervention seems like a promising strategy for reducing Internet addiction disorder, but more thorough research is needed to determine its effectiveness and suitability for use in a range of adolescent populations.

## Introduction and background

According to Young, a pattern of excessive or obsessive internet use that causes distress or impairment is known as Internet addiction (IA) [1]. There are several forms of IA, including computer addiction, information overload, net compulsions, cyber-sexual addiction, and cyber-relationship addiction [1]. In her Internet Addiction Test, Young modified the Diagnostic and Statistical Manual of Mental Disorders, fourth edition (DSM IV) criteria to relate to internet use and found that excessive internet use was most closely associated with pathological gambling, a disorder of impulse control in the DSM IV [1,2]. Despite the enormous number of people who use the Internet, the advantages are said to greatly exceed the negative effects of excessive use, but the IA, which is the consequence of uncontrolled use reportedly not yet recognized by the DSM-IV or the International Classification of Diseases (ICD-10) [3]. Various terms have been used to describe IA, including "Internet Addiction Disorder (IAD)," "Problematic Internet Use (PIU)," "Excessive Internet Use," "Compulsive Internet Use," "Pathological Internet Use," and "Computer Addiction." These terms all refer to the same idea, which is that a person become so engrossed in their online habits that they neglect other aspects of their lives [4-7].

Adolescence is the transitional period between childhood and adulthood according to developmental psychology, during which peer friendships are established, education is obtained, and social networks are expanded in preparation for future growth [8]. Adolescents go through rapid bio-psychosocial changes during this time. Adolescence is a time when emotional traits like restlessness, anxiety, timidity, and sudden excitement stand out. Because of these characteristics, modifications to an adolescent's living environment may pose a significant stressor, make them more difficult to manage, and have a detrimental effect on their social and mental growth [9]. Teenagers frequently use the internet for a variety of beneficial purposes, including entertainment, education, and communication. On the other hand, it is also known that prolonged use of the internet can lead to IA in addition to other negative effects like insomnia, pornography, and neglect of daily tasks [10]. IA is a significant issue among this age group. According to a meta-analysis, between 39% and 44% of teenagers in India suffer from smartphone addiction [11].

Adolescents with IAD may exhibit the following clinical symptoms: feeling obsessed with the internet, requiring more time to play games online, losing self-control, cutting back on or quitting internet use, feeling agitated, anxious, tense, moody, or depressed when attempting to limit internet time, and constantly using the internet to solve problems [12]. This may suggest that the internet has become a need and that using it can have addictive effects on users, harming their mental health and psychology. Adolescents’ compulsive behavior, fear of social interactions, loneliness, and habit of using the internet to solve problems are the main reasons why they use it excessively [13]. A meta-analysis revealed that the Middle East had the highest rate of IA (10.9%), followed by North America (8.0%) and Asia (7.1%). Among Asian teenagers, the prevalence of IA is 2.2-20.6% in China, 3.1-6.2% in Japan, 4.9-21.1% in the Philippines, and 3.0-16.4% in Hong Kong [14].

Mindfulness as a concept has been derived from Buddhist tradition; wherein it has been described as a central component of the thought process which helps in alleviating human suffering and is a way to bring about its cessation [15]. According to Neff and Costigan, mindfulness is the ability to observe one's experiences in the present moment without ignoring negative aspects of oneself or one's life [16]. It is also a significant predictor of well-being and life satisfaction [17,18]. The practice of mindfulness, which originated in Buddhist meditation, emphasizes maintaining a full, direct, and active awareness of psycho-spiritual phenomena experienced moment by moment [18]. 

Mindfulness is best described as a process of focusing one’s attention on the present situation and moment, with which an individual equips one through various forms of training like meditation, yoga, mental calmness and concentration exercises, breathing exercises, etc. With time such practices have also been adopted in the field of health sciences. As it offers an individual to have control over one’s thinking process it has proven a promising role in preventing and/or improving a wide group of mental and physical health conditions [19]. Mindfulness which has been found to have a modifiable effect on the intricate human mind, can be used as a health promotion intervention. Being a democratic, self-directed practice it can have greater acceptance and in turn benefit the individual, family, and community at large. While “mindfulness” has been defined as being aware of the present moment by paying attention purposefully and unfolding the experience, moment by moment, in a non-judgmental way; “mindfulness meditation” represents a systematic plan of action and framework, for cultivating this process into the daily regime in life by practice [20]. These two interventions, mindfulness-based stress reduction (MBSR) and mindfulness-based cognitive therapy (MBCT) integrate the essence of Eastern mindfulness practices into Western cognitive-behavioral practice.

Therefore, this narrative review aims to obtain greater insights into the effects of mindfulness-based interventions (MBI) on the mental health of adolescents with internet addiction.

## Review

Mindfulness-based interventions

A therapeutic strategy known as mindfulness intervention centers on developing nonjudgmental attention to thoughts, feelings, and physical sensations as well as present-moment awareness. It is frequently used to support emotional control and mental health by lowering stress, anxiety, and depression. Participants in MBIs, such as MBCT and MBSR, are encouraged to practice mindfulness techniques such as deep breathing, meditation, and mindful movement. Enhancing focus, building emotional resilience, and encouraging a closer connection to oneself and the environment are all benefits of these interventions [20].

MBSR was first developed and standardized for patients with chronic pain; it showed and demonstrated health benefits at both mental and body levels, thus helping people cope with many conditions [20]. The first and arguably most well-known mindfulness-based intervention to receive empirical support in the treatment of psychological symptoms is MBSR, which was developed by Kabat-Zinn in the early 1980s [21]. The goal of the eight-week MBSR treatment program is to reduce stress by improving mindfulness skills that are acquired through consistent meditation practice. The program includes daily audio-guided at-home meditation sessions (about 45 minutes per day), weekly two to two-and-half-hour group meditation classes taught by a qualified teacher, and a one-day mindfulness retreat that takes place in the sixth week. Learning to mindfully attend to body sensations through a variety of mind-body meditative techniques, including yoga, body scans, sitting meditation, and gentle stretching, is a major component of the course curriculum. In addition, the group classes promote conversation about applying these mindful practices in daily life, which eventually results in a more flexible approach to managing stress. The MBSR program was initially designed to treat medical patients who were experiencing chronic pain. Still, it has since been extended to include patients from a variety of medical and psychiatric backgrounds as well as community members. MBSR has been deemed generally tolerable by all of these diverse groups, with high rates of patient satisfaction, program completion, and compliance [21]. In a review, Kabat-Zinn mentioned that although MBI had an efficacy clinically, due to a lack of properly designed studies this could not be generalizable [21]. Hence there is still a growing need to substantiate these interventions in practice to build a foundation for the future. The author has put evidence on MBI in medicine and psychology in her review. The article sheds light on issues of cross-cultural sensitivity and how important it is for people who are teaching mindfulness to practice it themselves [21].

The most researched use of MBI is in MBCT, which was developed initially to prevent major depressive disorder relapses by Segal et al. [22]. As the name suggests, MBCT lessens the likelihood of recurrent depression by incorporating aspects of cognitive therapy (CT) and mindfulness training. The application of mindfulness principles helps people identify when their mood is declining without jumping to conclusions or taking action. Then, using this heightened awareness of oneself, the principles of cognitive therapy are combined to assist individuals in escaping the negative thought patterns that give rise to the symptomatology of depression. Except for this extra CT component, MBCT resembles MBSR in terms of format, consisting of an eight-week group-based program and homework assignments that vary in length and type. Since MBCT's inception, several carefully planned randomized-controlled trials comparing the treatment's effectiveness to control conditions have shown that the program is successful in lowering major depressive disorder patients' relapse rates [22]. A systematic review of 23 studies investigating the effects of MBSR and one study on both MBSR and MBCT, focusing on various aspects of employees' mental health, concluded that MBSR may help to improve their psychological health [23].

Application of mindfulness intervention in medical practice

In a review article by Raski, the author reviewed the literature on the health benefits of mindfulness and concluded that mindfulness has been found to promote subjective well-being, and has a preventive effect on pathologies related to stress and hypertension [24]. It was also found to enhance immune protection from viral and bacterial diseases. Mindfulness as an intervention affects both individual and interpersonal well-being. It has been found to positively impact stress, anxiety, and lifestyle choices. MBIs are effective in the treatment of skin problems like psoriasis, treatment of chronic pain, and psychiatric illnesses. Other than treatment the interventions were found to be effective in the promotion of empathy, compassion, and attentiveness. MBIs have also been found to improve the clinicians' quality of care, and mitigate work-related stress and burnout in healthcare workers [24].

In a review article by Chmielewski et al., they showed the role of MBI as a tool that could help healthcare professionals and medical students, to achieve a balance between work and rest, relieve stress, and in turn benefit their patients [25]. As per the authors, mindfulness training helped in assessing their mood perception, lowering stress, and responding to stimuli more effectively, which in turn had a positive impact on the service they rendered. The study also concluded that mindfulness meditation had a beneficial effect in combating stress, helped fight depression, prevent burnout, aided well-being, and helped achieve empathy among various categories of healthcare workers [25].

In another review by Ludwig and Kabat-Zinn on mindfulness in medicine, MBIs were identified as effective in improving conditions like depression, psychosis, stress, anxiety, insomnia, pain, hypertension, cancer-related symptoms, weight control, and addiction behaviors [26]. It was also shown to have an advantage in various workplaces, like healthcare settings, and schools [26]. In another systematic review by Billones and Saligan, MBIs are beneficial for medically unexplained symptoms [27].

Internet addiction disorder and its ill-effects

The internet is a useful resource for social interactions, work, education, and recreation. Additionally, it offers many benefits for developing young people's emotional intelligence [28]. Increased access to the internet has altered how people work, live, communicate, and learn, and it is now a crucial environment for their growth [29]. Using internet services in the education sector has improved teaching and learning in many ways. The ubiquitous accessibility of the Internet via smartphones and other gadgets is linked to various advantages, including the ability to obtain up-to-date information and a place for carrier planning and learning resources [30]. In universities, in particular, it has made it possible to remove geographical barriers and increase flexibility [31].

However, IA which is defined as a psychological dependence on the internet regardless of the activity one engages in after logging on, has many disadvantages [32,33]. Possible causes for this include: 1) prolonged sleep deprivation due to shifting from nighttime to daytime sleep; 2) melatonin secretion suppression from computer light exposure; and 3) increased sleep latency and decreased REM sleep from playing stimulating internet games, which raises catecholamine excretion and stimulates the sympathetic nervous system [33,34]. The unchecked, excessive, and compulsive use of the internet that results in psychological, social, and physical issues for the user is known as internet addiction. IA is characterized by an intense obsession with using the internet, trouble managing time on the internet, irritability when disturbed while online, and decreased social interaction in the real world and it is classified as a behavior addiction and impulse-control disorder [34,35]. Internet addiction is a new health issue in the technological age, as internet use has become commonplace due to scientific and technological advancements [35,36]. IA shares many characteristics with other behavioral addictions, including excessive use (craving for the internet), withdrawal (tension, anxiety, depression, and anger when the internet is unavailable for a few days), tolerance (increasing the amount of internet use required to feel satisfied), and negative outcomes (fatigue, poor time management, poor performance, deception, and social isolation) [36,37]. 

Due to historical and cultural variations, there are differences in the definition, diagnosis, and theories of internet addiction [37,38]. The access and use of the internet, smartphones, computers, and other electronic devices, has increased over the years; which has proved to be both a boon and a bane, as besides the benefits to the users, it has also led to negative health consequences. This problematic, compulsive use of the internet, reported from various countries, has now reached such a state, that it is being considered a significant public health concern. As a response measure, the World Health Organisation (WHO) has been conducting activities since 2014 on the public health-relevant issues with health conditions associated with the excessive use of the internet, including all other communications and gaming platforms [38]. Griffiths posits that internet addiction is a behavioral issue rather than a chemical one, negatively impacting day-to-day functioning because it allows users to access and remain in the online world whenever they choose, without being restricted by time or location [39]. In response to developments in research and the increasing demand for clinical treatment, the American Psychiatric Association (APA) has chosen to include "Internet Use Disorder" in the appendix of the fifth edition of the Diagnostic and Statistical Manual for Mental Disorders (DSM-V, APA, 2013) [40]. IA has been included in Appendix III of DSM-5, as an internet gaming disorder (IGD) [40].

In a review by Tripathi, the author reviewed many articles and very vividly summarized how IA and IGD have increased over the years, leading to personality disorders, and psychiatric problems [41]. Some of the ill effects discussed are low self-esteem, impulsive behavior, deterioration in sleep quality, mood disorders, and even extreme situations leading to suicides [41]. These effects of IA have been attributed to neuro-anatomical and neurochemical alterations in the brain leading to cortical thinning and an altered dopaminergic reward circuit. Due to the extensive neuropsychiatric and neurobiological effects of IA, several different therapeutic modalities have been required. It has been demonstrated that integrative therapy incorporating yoga and mindfulness is a successful tactic for treating many addiction disorders, including IA [41]. According to Chia et al., a study conducted in Southeast Asian countries revealed a noteworthy prevalence rate of 19.6% [42].

In a study by Jo et al., they compared DSM-5 IGD and the International Classification of Diseases (ICD-11) Gaming Disorder (GD) [43,44]. The authors have concluded, that their study was path-breaking and has provided empirical evidence that while ICD-11 emphasizes serious symptoms such as functional impairment caused by excessive internet use over a long time, it is different than DSM-5 [44]. According to Pan et al., there is an approximate prevalence of 7.02% for internet addiction, based on a systematic review and meta-analysis of studies conducted in 31 countries [45]. Numerous cross-sectional studies have demonstrated a strong correlation between increased symptoms of IA and depression, anxiety, stress, and insomnia. The majority of research has shown a strong correlation between insomnia and IA. People with IA are more likely to have sleep disorders and shorter sleep durations, according to a systematic meta-analysis [46]. Furthermore, longitudinal studies have demonstrated a positive correlation between the severity of anxiety symptoms and IA, as well as independent prediction of new IA incidence between baseline depression and anxiety status [47].

Additionally, there is a strong correlation between Obsessive Compulsive Disorder and anxiety, depression, and insomnia [48]. This is because teens who experience depression, anxiety, or stress in real life may use IA as an avoidance strategy by finding satisfaction in the virtual world [49]. Moreover, there is a chance that IA and mental health conditions will eventually overlap and interact. Prior research has demonstrated a significant correlation between mental health disorders and internet addiction. Furthermore, the earlier research highlighted the dangerous outcomes that underlie the co-occurrence of internet addiction and mental health issues, including a poorer prognosis, significant impairment to social functioning, and increased disruption to daily life [50]. According to some research, internet addiction is more common in young people and poses a risk to teenagers' ability to function and perform daily [51,52]. IA has been linked to significant issues with health, well-being, and family relationships, according to some studies [53]. According to a study done in six Asian nations, the prevalence of IA ranges from 5% to 21% [53]. In a systematic review, Wang et al. discovered a positive correlation between IA and attention-deficit/hyperactivity disorder (ADHD) in young adults and adolescents [54]. Clinically speaking, IA is taken seriously, and various nations have implemented different treatment modalities, confirming that individuals who experience hardships require expert assistance [55]. 

Furthermore, it has been discovered that using social applications, such as online instant messengers, social networking sites (SNSs), including Facebook, and online chatting, is linked to IA [56]. According to a study by Rabadi et al., the amount of time spent online showed unique usage patterns [57]. Participants who were internet addicted reported higher rates of online shopping, community activities, and gaming, while those who were not addicted reported higher rates of information searches, email use, and chatter [57].

IAD is becoming more and more of a mental health issue, especially for teenagers, according to recent research [58]. Recent advances in neuroscience demonstrate important alterations in brain morphology and function linked to internet addiction. The modification of the brain's functional connectivity, particularly in the reward and cognitive control networks, is one important finding. For instance, the anterior cingulate cortex and dorsolateral prefrontal cortex, which are essential for emotion regulation and inhibitory control, show reduced activity in adolescents with IAD. These modifications could account for the impetuous actions frequently displayed by those who are battling IA [58]. In addition, novel predictive models based on connectomes have been utilized to gain a deeper comprehension of the neurobiological foundations of IAD. These models provide insights that may enhance targeted treatments by connecting specific brain connectivity patterns to behaviors linked to IA. Large-scale brain networks, including the fronto-parietal and default mode networks, which are essential for self-control and decision-making, have been shown to have imbalances by researchers [59].

IAD prevalence is still very different in different parts of the world. Research indicates that IA rates can reach 47.4% in Asia, whereas rates in Europe vary from 1.6% to 5.5%, contingent upon the nation and the diagnostic standards applied. Variations in internet access, cultural influences, and evaluation techniques are probably the causes of the variability [60].

In 2024, new research on IAD has produced several interesting discoveries [61,62]. According to a study on adolescent brain imaging, IAD alters the functional connectivity of brain networks, particularly the default mode network, which is important for attention and introspection. People may find it more difficult to control their internet use and emotional reactions if there are disruptions in these brain regions, which may also have an impact on behavior and cognitive development. These results imply that IA may have long-term consequences for mental health, particularly for growing brains [61]. Studying genetic effects in addition to neurobiological effects has advanced our understanding of IAD. An association between IA and the serotonin receptor gene HTR2A polymorphism (rs6313) was found in a 2024 study [62]. Because this genetic variation affects impulse control and emotional regulation, people who have it are more prone to IAD. These discoveries may pave the way for customized preventative measures, such as targeted CBT and genetic screening [62].

The treatment of IA is multimodal. It includes both non-psychological and psychological approaches. Non-psychological approaches include sports, physical exercise (which can compensate for the decrease of the dopamine level), and pharmacological (selective serotonin-reuptake inhibitors, non-tricyclic antidepressants, psychostimulant drugs, etc). Psychological approaches include reality therapy, acceptance, and commitment therapy, interpersonal group psychotherapy, motivational interviewing, MBSR, mindfulness-based relapse prevention, cognitive-behavioral therapy, multi-level counseling programs, individual psychotherapy, and community reinforcement and family training. Counseling on life balance, life visioning, education about addiction, training in assertiveness and communication, social skills, and life skills all help to support these approaches. An organized aftercare procedure comes next. Following treatment entails keeping an eye on how technology is used, continuing group and psychotherapy sessions, and outpatient treatment that is personalized and comprehensive. Together, these strategies have shown to be successful in many research projects [63].

Due to sociocultural and socioeconomic factors, the diagnostic criteria and classification of IAD remain controversial, and its assessment instruments are inconsistent. Some academics contend that defining the IAD's boundaries based on conventional addiction criteria obscures the distinctive characteristics of behavior. There have been suggestions that IAD is a problematic behavior rather than a mental illness or addiction. Its clinical characteristics and underlying theories are still being researched and updated as of right now.

Internet addiction disorder and adolescence

Adolescence is the time between childhood and adulthood. It is a time when people feel more at ease expressing their emotions to their peers than to their parents and when their emotional regulation is still developing. With this process of transformation, it is evident that the person has many questions about his future and himself. Adolescence is a time when people are most susceptible to the allure and complexity of social environments, which can lead to many risky behaviors for their health [64].

The population group with the highest prevalence of internet use is adolescents. Teenagers use the internet as a place to expand on the identity they are creating. In actuality, it's a place where they congregate free from parental supervision and mold to their standards, using a common language and set of preferences. The teenager might experience a sense of identification and group belonging in this area. The teenager will also be able to create virtual personas on the internet, or avatars, that they can customize to project the image they want. The adolescent's process of constructing their identity includes this virtual construction, which allows them to experiment with being whoever and whatever they want to be around their peers [65].

IA is positively correlated with chatting, blogging, online shopping, using porn, and other activities in addition to gaming [66]. It has been demonstrated that adolescents from divorced homes are more prone to experience IA [67, 68]. Teens with IA were more likely to have divorced parents, and the bulk of them were men, according to a study done on Taiwanese teenagers [68]. The discovery was that after switching to online classes, students with low grade point averages had higher scores for IA [69].

Nonetheless, as students' academic performance improves, the severity of IA will decline [70]. Teenagers using the internet as a coping mechanism for real-world stress and increased academic stress are the reasons behind the relationship between online learning and IA [71]. Nowadays, with nearly all daily activities taking place online, teens naturally develop an insatiable desire to stay online to use games and social media to lift their spirits. This leads to a host of other symptoms associated with IA [72].

Helena Hamu et al. outlined how dopamine levels in the adolescent brain are higher in the prefrontal cortex but lower in the pleasure center (nucleus accumbens) [73]. Teens may become more excited and euphoric about something if their dopamine levels are lowered because the dopamine release pathway is blocked in chronic IA. As a result, adolescents find it simpler to turn such behavior into an addiction [74]. Internet and smartphone addiction scores, as well as depression and anxiety scores, were positively correlated with both brain parenchyma γ-aminobutyric acid (BP-GABA) and grey matter γ-aminobutyric acid (GM-GABA) [75]. According to a study conducted on 467 Chinese teenagers, people with IAD or IGD may participate in riskier activities like skipping class, smoking, drinking, fighting, gambling, stealing, and so forth [76]. Adolescents with depression could access supportive social networks and a sense of control in a virtual world on the internet, allowing them to ignore their emotional struggles and escape reality.

According to research by Yayan et al., teenagers who exhibited IA symptoms in real life had poorer interpersonal relationships or nervous social phobias [77]. Teenagers frequently use the internet to relieve their mental health issues, like anxiety, by engaging in enjoyable activities, unwinding on social media, and anonymously venting. There has been positive research on the relationship between adolescents and IAD. Risky lifestyle choices including drug abuse, alcoholism, smoking, suicidal thoughts and actions, and gambling are strongly linked to the development of risky behaviors in adolescents [77].

Role of mindfulness intervention in the management of internet addiction

MBIs have been applied in behavioral addiction studies in recent years, and have been shown to have a direct and indirect impact on the excessive usage of the internet. Gordon et al., in their write-up for an editorial on mindfulness and behavioral addiction, mention that mindfulness as an intervention has proved beneficial in all sorts of addictions, stating a few are gambling, substance abuse, and workaholic [78]. Later the authors recommended that MBIs can also be used in other forms of behavioral addiction like IA, social networking addiction, sex addiction, video game addiction, etc. There have been improvements in sleep quality, psychological parameters, and work performance post-intervention [78]. A study by Arslan et al., among undergraduate students concluded that MBIs can help design prevention strategies for reducing internet-related addictive behaviors [79].

MBIs can also help to reduce the internet's addictive behavior. In a study by Lee et al. on “the effect of MBIs on IA among college students”, the intervention measure helped reduce IA in nearly 87.5% of the participants [80]. It has also helped in increasing self-awareness and developing self-control over the time they spend on the internet. It also helped in improving their sleep quality and sleep duration. The study concluded that Mindfulness can be used as an effective intervention measure to decrease IA symptoms and help improve not only psychological health but also life quality [80].

In another study among undergraduate students mindfulness-based cognitive-behavioral intervention proved beneficial in decreasing smartphone addictions [81]. A study on adolescents by Liu et al., using logotherapy-based mindfulness intervention was found to reduce the IA significantly during the COVID-19 period and helped improve their positive emotions, thereby reducing their negative emotions, and alleviating the degree of anxiety and depression [82]. Recent developments in psychotherapy theories from the standpoint of positive psychology include logotherapy and mindfulness therapy [82]. A review article of 18 studies by Sharma et al. on whether MBIs were beneficial in the case of IGD, showed it to help promote emotional regulation and metacognitive awareness [83]. It was also seen to help develop adaptive coping/cognition and reduce the impulsivity and craving for playing games [83]. Another review article by Sun emphasized how mindfulness training sessions should be adopted by the school, community, and working environment to combat the negative effects of excessive use of social media; IA and its negative effects like sleep deprivation, psychological problems, and lowered study and working efficiency, can thus be decreased [84].

Adolescent populations have received MBIs in the treatment and resilience-building contexts, including as a component of extensive health promotion programs. For example, studies conducted in treatment settings demonstrate that mindfulness can reduce symptoms of (but are not limited to) (a) teenage anxiety and depression; (b) intrusive thoughts, hostile thoughts, negative coping, rumination, and emotional arousal; (c) co-occurring substance use disorder and post-traumatic stress disorder; and (d) diabetes [85-87]. Participants learn to increase their perception of distance from mental urges through mindfulness techniques. The following factors have led to the decision that this method is appropriate for treating behavioral addictions: (a) relapse and withdrawal symptoms can be lessened by meditation; (b) mindfulness can control an emotional state of distress related to addiction; (c) the techniques can assist in appreciating life's intrinsic value rather than the fleeting pleasure of addictive activities; (d) salience can be lessened; and (e) patience can be enhanced [88].

Recently, mindfulness techniques have been used to treat a variety of mental illnesses, including behavioral addiction [89-91]. A few academics have examined the viability of MBI and confirmed its impact on IA [92]. By quantitatively assessing mindfulness and examining its connection to IA, some studies have even provided insights into the workings of this kind of intervention [92].

Discussion

According to the results of this narrative review, adolescents with IAD may benefit greatly from MBIs. The reviewed literature consistently shows that by improving emotional regulation, attention, and self-awareness, mindfulness practices such as breathing exercises, meditation, and mindful awareness techniques can lessen compulsive internet use. These strategies are essential for controlling the impulsivity, tension, and anxiety that frequently accompany teenagers' excessive internet use. The literature's main conclusions are that mindfulness practices help people become more conscious of their routines and thought processes. This enables them to identify the situations and people that set off binge internet use. Due to their developmental stage and high level of digital technology use, adolescents are especially prone to IA. By learning to respond more mindfully to emotional stressors instead of running away into virtual worlds, adolescents may benefit from MBIs.

The review also emphasizes the indirect benefits of MBIs on co-occurring mental health conditions like stress, anxiety, and depression, which frequently make IAD worse. Teens with these underlying illnesses might use the internet to get short-term relief, but mindfulness provides longer-term, more beneficial coping strategies. According to many studies, teens who practice mindfulness report increases in overall psychological well-being, improved emotional regulation, increased focus, and improved cognitive flexibility in addition to decreased internet usage. Notwithstanding the encouraging results, the review also highlights certain shortcomings in the existing corpus of literature. With small sample sizes and scant longitudinal data, the majority of studies on mindfulness and IAD in teenagers are preliminary. Because of this, it is challenging to extrapolate the results and assess the interventions' long-term effectiveness. Furthermore, it is difficult to create standardized protocols that can be widely used due to the diversity of mindfulness practices and the variations in program delivery (duration, intensity, and modality).

A further drawback is the paucity of research comparing MBIs to other well-researched therapies for IAD, like CBT. Although mindfulness seems promising, more studies are required to compare its efficacy with other interventions and determine how mindfulness fits into the overall treatment plan for IAD. Given these results, larger and more diverse adolescent populations should be the focus of future research, and long-term follow-up studies should be carried out to evaluate the sustainability of MBIs for IA. Furthermore, creating standardized mindfulness programs especially designed to handle the particular difficulties presented by IAD may enhance treatment results. Adolescent IAD prevention and management may also be made more approachable and proactive by incorporating these interventions into community programs or school curricula.

In summary, MBIs offer a viable, affordable, and easily obtainable strategy for reducing the symptoms of IAD in teenagers. Although further research is required to determine the long-term effectiveness and standardize protocols, the potential advantages for both IAD and general mental health highlight the significance of integrating mindfulness into prevention and treatment plans for teenagers who are addicted to the internet.

Implications

This narrative review has broad implications regarding the impact of MBIs on adolescent IAD. First, the review emphasizes how mindfulness-based therapies can be a useful non-pharmacological strategy for controlling and mitigating IAD symptoms. Adolescents can benefit from MBIs by developing better attention spans, self-awareness, and emotional control, all of which can lessen compulsive online behaviors and help them form healthier relationships with the internet. According to this review, incorporating mindfulness practices into therapeutic or educational programs may help adolescents learn coping mechanisms for handling stress, anxiety, and impulsivity which are frequent causes of binge internet use. Beyond IA, the application of mindfulness programs may also improve mental health outcomes, such as lowering anxiety and depression, which frequently co-occur with IAD.

Given the increasing apprehension regarding digital dependency, this review proposes additional empirical research to develop standardized mindfulness protocols designed especially for adolescents with IAD. To address the rising incidence of youth IA, policymakers, and mental health professionals ought to think about integrating MBIs into public health strategies. In the end, MBIs offer a potential means of cultivating a healthy and balanced digital environment for the younger demographic.

## Conclusions

This review was an attempt to identify if and how mindfulness training sessions can be adopted as an intervention in the management of internet addiction. Mindfulness as an intervention in health sciences research has found various applications. Being democratic and self-directed has been found to have better acceptance by individuals, their families, and the community at large. Other than treatment of pathological conditions like psoriasis, chronic pain, psychiatric conditions, mindfulness has also been found to promote subjective well-being, and prevent stress and hypertension, enhance immune protection against various viral and bacterial conditions. With the changing lifestyle, easy access to smartphones and digital devices, and more indulgence of people on social media and networking platforms, internet addiction has been on a rising trend over the last two decades. Activities on public health-relevant issues and associated health conditions related to excessive use of the internet, including gaming have been taken as a response measure by the World Health Organization.

Internet addiction and its ill effects like low self-esteem, impulsive behavior, deterioration of sleep quality, and mood disorders, are a growing concern. The treatment of internet addiction is multimodal-non-psychological (sports, physical exercise), pharmacological (including serotonin-receptor inhibitors, non-tricyclic antidepressants, etc.), and psychological (mindfulness-based stress reduction, cognitive behavioral therapy). Mindfulness intervention (MBI) and its role in internet addiction have been tried in various places and populations. MBIs are beneficial as per studies done, where it has been found to have been applied in behavioral addiction studies, in studies designed to prevent internet-related addictive behavior, reduction of internet addiction, decrease smartphone addictions, improve positive emotions, and decrease anxiety and depression, effective in combating internet gaming disorder. Thus MBIs can be an effective tool in designing strategies for the prevention and treatment of internet addiction.
